# Colorectal Perforation in Patients with Connective Tissue Disease

**DOI:** 10.1155/2019/5852438

**Published:** 2019-06-19

**Authors:** Kiichi Sugimoto, Kazuhiro Sakamoto, Yu Okazawa, Rina Takahashi, Kosuke Mizukoshi, Hisashi Ro, Masaya Kawai, Shingo Kawano, Shinya Munakata, Shun Ishiyama, Hirohiko Kamiyama, Makoto Takahashi, Yutaka Kojima, Yuichi Tomiki, Naoto Tamura

**Affiliations:** ^1^Department of Coloproctological Surgery, Juntendo University, Faculty of Medicine, Tokyo 113-8421, Japan; ^2^Department of Rheumatology, Juntendo University, Faculty of Medicine, Tokyo 113-8421, Japan

## Abstract

**Purpose:**

The goal of this retrospective study was to identify prognostic factors associated with mortality after surgery for colorectal perforation among patients with connective tissue disease (CTD) and to review postoperative outcomes based on these prognostic factors.

**Methods:**

The subjects were 105 patients (CTD group: n=26, 24.8%; non-CTD group: n=79, 75.2%) who underwent surgery for colorectal perforation at our department. Cases with iatrogenic perforation due to colonoscopic examination were excluded from the study. We retrospectively investigated perioperative clinicopathological factors in patients undergoing surgery for colorectal perforation.

**Results:**

There were 7 patients (6.7%) who died within 28 days after surgery in all patients. In multivariate analysis, CTD and fecal peritonitis emerged as significant independent prognostic factors (p=0.005, odds ratio=12.39; p=0.04, odds ratio=7.10, respectively). There were 5 patients (19.2%) who died within 28 days after surgery in the CTD group. In multivariate analysis, fecal peritonitis emerged as a significant independent prognostic factor in the CTD group (p=0.03, odds ratio=31.96). The cumulative survival curve in the CTD group was significantly worse than that in the non-CTD group (p=0.006). An analysis based on the presence of fecal peritonitis indicated no significant difference in cumulative survival curves for patients without fecal peritonitis in the CTD and non-CTD groups (p=0.55) but a significant difference in these curves for patients with fecal peritonitis in the two groups (p<0.0001).

**Conclusions:**

This study demonstrated that cumulative survival in patients with CTD is significantly worse than that in patients without CTD after surgery for colorectal perforation.

## 1. Introduction

Connective tissue disease (CTD) is chronic, inflammatory, autoimmune disorders that manifest clinically in multiple ways in vascular and connective tissue [1]. The pathogenesis of these conditions is thought to involve deposition of immune complexes in blood vessel walls, which may produce thrombosis, ischemic changes and colitis due to effects on blood supply to the colon and rectum [1]. Therefore, CTD has many gastrointestinal manifestations, including obstruction, hemorrhage, ischemia, and perforation [1, 2]. In addition to the diseases themselves, administered steroids and other drugs, including nonsteroidal anti-inflammatory drugs (NSAIDs), may play a causative role in colorectal disorders such as colonic diverticular perforation [3]. These drugs often mask nonspecific symptoms in patients with CTD [2], and this can result in a delay of treatment after onset of the colorectal disorder. Consequently, CTD has high mortality since patients often present with colorectal perforation, bowel ischemia, and collagen colitis [1, 4]. In particular, colorectal perforation is a life-threatening disease since it may lead to septic shock and multiple organ failure (MOF) [5–8], and this may explain the very high mortality among patients with CTD [2]. Therefore, it is crucial to understand the characteristics of colorectal perforation in patients with CTD to allow effective treatment. For these reasons, we retrospectively identified prognostic factors associated with mortality after surgery for colorectal perforation, with subsequent comparison of cumulative survival curves based on the identified prognostic factors.

## 2. Materials and Methods

### 2.1. Patient Selection

The subjects were 105 patients who underwent surgery for colorectal perforation at our department between January 2003 and September 2017 (i.e., the past 15 years). Cases with iatrogenic perforation due to colonoscopic examination were excluded from the study.

### 2.2. Clinicopathological Factors

We retrospectively investigated perioperative clinicopathological factors in patients undergoing surgery for colorectal perforation. Patients who died within 28 days after surgery were classified as nonsurvivors. Clinicopathological factors of patient background, preoperative status, surgical factors, and postoperative status were analyzed. The patient background included age, gender and preoperative comorbidities including CTD. Preoperative status included white blood cell count (WBC) (/mm^3^), C-reactive protein (CRP) (mg/dl), systemic inflammatory response syndrome (SIRS), dose of steroids per day (mg/day; equivalent to prednisolone), accumulated steroid dose per month (mg/month; equivalent to prednisolone), duration of CTD (years), duration of steroid intake (years), steroid pulse therapy and immunosuppressive agent. Surgical factors included the time from onset of symptoms to operation (>12 hr/≤12 hr), perforation site (Cecum (C)-Transverse colon (T)/Descending colon (D)-Rectum (R)), and presence of fecal peritonitis. Peritonitis was classified according to the modified Hinchey grading system [9], which simply distinguishes fecal peritonitis from purulent peritonitis without considering the degree and extent of fecal peritonitis. Postoperative status included WBC on postoperative day (POD) 1 (/mm^3^), CRP on POD 1 (mg/dl), positivity of blood culture (not performed in all patients), presence of disseminated intravascular coagulation (DIC), direct hemoperfusion with polymyxin B immobilized fiber (PMX-DHP), and postoperative dose of steroids per day (mg/day; equivalent to prednisolone).

### 2.3. Therapeutic Strategy for Colorectal Perforation

We have described our therapeutic strategy for colorectal perforation elsewhere [6]. Briefly, emergency surgery should be performed immediately, but it is preferable for patients with concomitant septic shock to receive anti-shock therapy prior to surgery. The causative bacteria are a determinant of the clinical course of patients with septic shock, and these bacteria should be identified in blood culture performed after surgery. With respect to the use of antibiotics, cephem antibiotics are mainly used immediately after diagnosis of colorectal perforation. For a case that is refractory to cephem antibiotics, carbapenem antibiotics or quinolone is used based on the results of cultures of intraperitoneal abscess fluid or blood. Crystalloid fluid solution is mainly used to maintain circulatory blood volume and an optimum urine output of 0.5-1.0 ml/kg/h. For a case with low output, in which infusion of crystalloid solution is insufficient to maintain blood pressure, noradrenaline or another vasopressor is also given in a continuous manner. Postoperative respiratory failure is handled with mechanical ventilation via intratracheal intubation, and tracheotomy may be required if this condition persists. For patients who develop DIC after surgery, recent therapeutic strategies use heparin, gabexate mesylate, nafamostat mesilate and trombomodulin *α*, and antithrombin III is used for patients with decreased antithrombin activity, although this approach has changed over time. We used these standard strategies throughout the study period.

### 2.4. Statistical Analysis

Discrete variables were compared using a Fisher exact probability test and continuous variables were compared by Mann-Whitney U-test. Clinicopathological factors that showed a significant difference or trend in univariate analysis were used as covariates in multivariate analysis using a logistic regression model and a stepwise procedure, with the odds ratio used as a measure of association. The Kaplan-Meier method was used to calculate cumulative survival, and univariate analyses were performed by log-rank test. Differences were considered significant at p < 0.05. Values are expressed as median (minimum-maximum).

## 3. Results 

### 3.1. Clinicopathological Factors in Survivors and Nonsurvivors among All Patients

There were 7 patients (6.7%) in the nonsurvivor group (Table 1). In univariate analysis, CTD, fecal peritonitis and postoperative DIC were significantly more common in this group compared with the survivor group (p=0.01, 0.04, and 0.03, respectively). No other clinicopathological factors differed significantly between the two groups. In multivariate analysis using these three factors, CTD and fecal peritonitis emerged as significant independent prognostic factors (p=0.005, odds ratio=12.39; p=0.04, odds ratio=7.10, respectively) (Table 2).

### 3.2. The Characteristics of the Patient with CTD

Patient characteristics are shown in Table 3. The 26 patients with CTD had a median age of 61.5 (28-95) years, and included 7 males (26.9%) and 19 females (73.1%). CTD was due to systemic lupus erythematosus (SLE) (n=6, 23.1%); polyarteritis nodosa (PN) (n=5, 19.2%); mixed connective tissue disease (MCTD) (n=4, 15.4%); malignant rheumatoid arthritis (MRA), rheumatoid arthritis (RA), and polymyalgia rheumatica (PMR) (each n=2, 7.7%); and dermatomyositis (DM), systemic sclerosis (SSC), adult Still disease, Sjögren syndrome, and Wegener granulomatosis (each n=1, 3.8%). There was no patient with Ehlers-Danlos syndrome. The most frequent perforation site was the sigmoid colon (n=8, 30.8%) and the most common cause of perforation was diverticulum (n=13, 50.0%).

### 3.3. Clinicopathological Factors in Patients with and without CTD

When the differences of the clinicopathological factors in patients between with and without CTD were analyzed, in univariate analysis, there were significantly more females (p=0.01) and higher rates of positive blood culture (p<0.001) and DIC after surgery (p=0.03) in patients with CTD (CTD group) compared to those without CTD (non-CTD group) (Table 4). No other clinicopathological factors differed significantly between these groups.

### 3.4. Clinicopathological Factors in Survivors and Nonsurvivors among Patients with CTD

There were 5 patients (19.2%) in the nonsurvivor group (Table 5). In univariate analysis, fecal peritonitis was significantly more common in this group compared with the survivor group (p=0.01), and there was a trend for a shorter duration of CTD in the nonsurvivor group (p=0.055). No other clinicopathological factors differed significantly between the two groups. In multivariate analysis using these two factors, fecal peritonitis emerged as a significant independent prognostic factor (p=0.03, odds ratio=31.96) (Table 6). Detailed information for the five nonsurvivors within 28 days after surgery is shown in Table 7.

### 3.5. Cumulative Survival Curves in Patients with and without CTD

**Figure 1:**
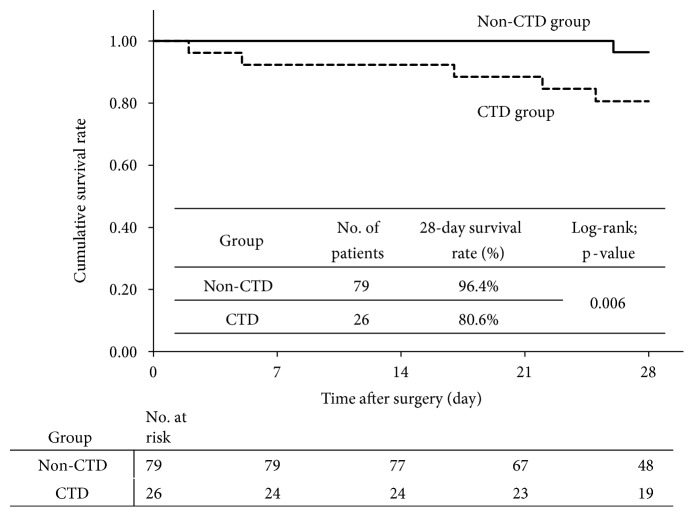
Cumulative survival curves based on the presence of connective tissue disease (CTD).

**Figure 2:**
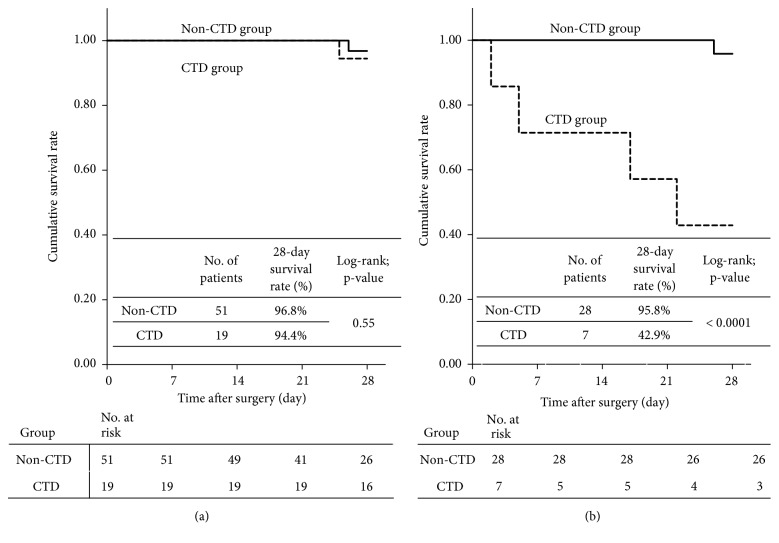
Cumulative survival curves based on the presence of connective tissue disease (CTD) in (a) patients without fecal peritonitis and (b) patients with fecal peritonitis.

The cumulative survival curve in the CTD group was significantly worse than that in the non-CTD group (28-day survival rate: 80.6% vs. 96.4%, p=0.006 by log-rank test) (Figure 1). An analysis based on the presence of fecal peritonitis indicated no significant difference in cumulative survival curves for patients without fecal peritonitis in the CTD and non-CTD groups (p=0.55) (Figure 2(a)), but a significant difference in these curves for patients with fecal peritonitis in the two groups (p<0.0001) (Figure 2(b)).

## 4. Discussion

In this study, the coexistence of CTD and the presence of fecal peritonitis were found to be significant independent prognostic factors among all patients. Moreover, the presence of fecal peritonitis emerged as the only independent prognostic factor for survival in patients with CTD. Fecal peritonitis due to colorectal perforation has previously been reported to have a huge impact on high mortality [10, 11]. Bacteremia can be caused by Gram-negative bacteria such as* Escherichia coli*, and endotoxins on the outer membranes of these bacteria interact with the host in Gram-negative sepsis [12]. These endotoxins may also cause septic shock and MOF through release of cytokines such as interleukin-1 and tumor necrosis factor-*α* [13]. Patients with CTD, who essentially have immunosuppressed backgrounds, are more likely to be vulnerable to devastating conditions caused by peritonitis due to colorectal perforation, which can lead to mortality. However, if fecal peritonitis is well managed, it does not significantly influence mortality [10]. In addition, since fecal peritonitis is usually a polymicrobial infection with a high bacterial burden, recent recommendations state that delaying antimicrobial treatment in patients with sepsis can be associated with high mortality [14]. Therefore, early surgical intervention with appropriate antibacterial therapy appears to be crucial to improve outcomes in patients with CTD.

Contrary to our expectations, nonsurvivors had a shorter period of CTD than survivors. However, this might be because nonsurvivors with a shorter period of CTD also had fecal peritonitis, as shown in Table 7. As mentioned above, fecal peritonitis is a powerful prognostic factor. Therefore, the period of CTD may not matter if fecal peritonitis occurs in a patient with CTD. However, there were only five patients with CTD who died in our study, and a further study in more patients is required to draw a conclusion on this issue.

In a study of risk factors in abdominal surgery for patients with CTD, Nakashima et al. [2] found that a higher dose of steroids administered at the time of surgery was significantly associated with higher mortality. Our findings are not consistent with this result. This may be because Nakashima et al. included both elective and emergency operations for any type of gastroenterological disease, rather than procedures limited to colorectal perforation. Indeed, corticosteroids can induce an immunosuppressive condition that can lead to sepsis in patients with CTD [15, 16], and inflammatory reactions are easily masked by corticosteroids [17]. It is evident that early diagnosis and prompt initiation of surgical intervention and antibacterial treatment is essential to improve the prognosis of patients with CTD who develop sepsis due to colorectal perforation. Therefore, despite our results, very careful monitoring is required when colorectal perforation is suspected in patients with CTD.

The CTD group in the current study included more female patients and more patients with a positive blood culture and DIC after surgery, compared with the non-CTD group. CTD is generally known to be more common in women. With respect to the positivity of blood culture, our findings may reflect an immunosuppressive condition in patients with CTD. However, it was interesting that the positivity of blood culture did not affect mortality in patients with CTD. Since blood culture was not performed in all patients in this study, further investigation in more patients with CTD may be needed to examine this issue. With respect to DIC, since CTD itself or administered steroids can cause an immunodeficient status, it is natural that patients with CTD can develop DIC after surgery for colorectal perforation. Originally, a variety of abnormalities of coagulation mechanisms were associated with patients with SLE [18, 19], which suggests that patients with CTD may be more likely to develop DIC due to organ failure. In our study, there was a significant difference in cumulative survival curves between the CTD and non-CTD groups in patients with fecal peritonitis. This suggests a reduced capacity for recovery from fecal peritonitis in patients with CTD based on their immunosuppressive condition. Therefore, patients with CTD who develop fecal peritonitis due to colorectal perforation require prompt treatment. Polymyxin B hemoperfusion has recently been reported to be effective for particularly severe sepsis or septic shock due to abdominal infections [20]. Since our study demonstrated that patients with CTD were more likely to develop life-threatening conditions, such a treatment strategy should be considered for these patients.

Finally, there are several limitations in this study that are inherent to retrospective studies. Firstly, the data were collected at a single hospital and only a small number of patients were enrolled. Therefore, a validation study in a larger cohort is required. Secondly, we did not have detailed information with respect to the use of NSAIDs. Within these limitations, we conclude that cumulative survival in patients with CTD is significantly worse than that in patients without CTD after surgery for colorectal perforation and that patients with CTD who develop fecal peritonitis due to colorectal perforation should be treated promptly.

## 5. Conclusions

This study demonstrated that cumulative survival in patients with CTD is significantly worse than that in patients without CTD after surgery for colorectal perforation and that patients with CTD who develop fecal peritonitis due to colorectal perforation should be treated promptly.

## Figures and Tables

**Table 1:** Clinicopathological factors in survivors and non-survivors among all patients.

	Survivor group (n=98)	Non-survivor group (n=7)	p-value
1. Patients backgrounds			

Age *∗*	62 (18 - 94)	73 (28 – 95)	0.09
Gender (Male/Female)	47 (48.0%)/51 (52.0%)	4 (57.1%)/3 (42.9%)	0.71
Preoperative comorbidity ^†^			
Neurological disease (+/-)	11 (11.2%)/87 (88.8%)	1 (14.3%)/6 (85.7%)	0.58
Cardiovascular disease (+/-)	14 (14.3%)/84 (85.7%)	0 (0%)/7 (100%)	0.59
Respiratory disease (+/-)	12 (12.2%)/86 (87.8%)	0 (0%)/7 (100%)	1.00
Gastroenterological disease (+/-)	13 (13.3%)/85 (86.7%)	2 (28.6%)/5 (71.4%)	0.26
Renal disease (+/-)	10 (10.2%)/88 (89.8%)	2 (28.6%)/5 (71.4%)	0.18
Hematological disease (+/-)	3 (3.1%)/95 (96.9%)	0 (0%)/7 (100%)	1.00
Endocrinological disease (+/-)	11 (11.2%)/87 (88.8%)	1 (14.3%)/6 (85.7%)	0.58
Diabetes Mellitus (+/-)	17 (17.3%)/81 (82.6%)	3 (42.9%)/4 (57.1%)	0.13
Hypertension (+/-)	24 (24.5%)/74 (75.5%)	1 (14.3%)/6 (85.7%)	1.00
Collagen tissue disease (CTD) (+/-)	21 (21.4%)/77 (78.6%)	5 (71.4%)/2 (28.6%)	0.01

2. Preoperative status			

WBC (/mm^3^) *∗*	8950 (1000 - 28800)	7700 (1100 - 37400)	0.83
CRP (mg/dl) *∗*	12.0 (0.1 - 48.9)	16.9 (3.0 - 32.0)	0.12
SIRS (+/-)	49 (50.0%)/49 (50.0%)	5 (71.4%)/2 (28.6%)	0.44

3. Surgical factors			

The time from onset of symptoms to operation (> 12hr/≤12hr)	56 (57.1%)/42 (42.9%)	3 (42.9%)/4 (57.1%)	0.70
Perforation site (C-T/D-R)	28 (28.6%)/70 (71.4%)	1 (14.3%)/6 (85.7%)	0.67
Fecal peritonitis (+/-)	30 (30.6%)/68 (69.4%)	5 (71.4%)/2 (28.6%)	0.04

4. Postoperative status			

WBC on POD 1 (/mm^3^) *∗*	8750 (700 - 40300)	5600 (900 - 39900)	0.19
CRP on POD 1 (mg/dl) *∗*	15.6 (0.2 - 37.8)	12.7 (6.6 - 27.1)	0.72
Blood culture (+/-) ^††^	24 (32.9%)/49 (67.1%)	1 (20.0%)/4 (80.0%)	1.00
DIC (+/-)	28 (28.6%)/70 (71.4%)	5 (71.4%)/2 (28.6%)	0.03
PMX-DHP (+/-)	34 (34.7%)/64 (65.3%)	5 (71.4%)/2 (28.6%)	0.12

*∗* Median (min-max), ^†^ With some duplications, ^††^ Blood culture was carried out in 78 patients

WBC: white blood cell. CRP: C-reactive protein. SIRS: systemic inflammatory response syndrome. C-T: Cecum - Transverse colon. D-R: Descending colon - Rectum. POD: postoperative day. DIC: disseminated intravascular coagulation. PMX-DHP: polymyxin B-immobilized fiber column hemoperfusion.

**Table 2:** Prognostic factors for mortality within 28 days after surgery in all patients.

	p-value	Odds ratio	95% Confidence interval
Collagen tissue disease (CTD)	0.005	12.39	2.07 - 108.82
Fecal peritonitis	0.04	7.10	1.11 - 64.82
Postoperative DIC	0.29	2.69	0.42 - 22.03

DIC: disseminated intravascular coagulation.

**Table 3:** The Characteristics of the patient with CTD.

	No. of patients	(%)
Total	26	
Age **∗**	61.5	(28-95)
Sex		
Male	7	26.9%
Female	19	73.1%
Connective tissue disease (CTD)		
Systemic lupus erythematosus (SLE)	6	23.1%
Polyarteritis nodosa (PN)	5	19.2%
Mixed connective tissue disease (MCTD)	4	15.4%
Malignant rheumatoid arthritis (MRA)	2	7.7%
Rheumatoid arthritis (RA)	2	7.7%
Polymyalgia rheumatica (PMR)	2	7.7%
Dermatomyositis (DM)	1	3.8%
Systemic sclerosis (SSC)	1	3.8%
Adult Still disease	1	3.8%
Sjögren's syndrome	1	3.8%
Wegener granulomatosis	1	3.8%
Perforation site		
Cecum	3	11.5%
Ascending colon	4	15.4%
Transverse colon	3	11.5%
Descending colon	3	11.5%
Sigmoid colon	8	30.8%
Rectosigmoid colon	4	15.4%
Rectum	1	3.8%
Cause of perforation		
Diverticulum	13	50.0%
Idiopathic	5	19.2%
Ulcer	5	19.2%
Ischemic	1	3.8%
Colorectal cancer	1	3.8%
Cytomegalovirus enteritis	1	3.8%

*∗* Median (min-max).

**Table 4:** Clinicopathological factors in patients with and without connective tissue disease (CTD).

	CTD group (n=26)	Non-CTD group (n=79)	p-value
1. Patients backgrounds			

Age **∗**	61.5 (28 - 95)	63 (18 – 94)	0.70
Gender (Male/Female)	7 (26.9%)/19 (73.1%)	44 (55.7%)/35 (44.3%)	0.01
Preoperative comorbidity ^†^			
Neurological disease (+/-)	1 (3.8%)/25 (96.2%)	11 (13.9%)/68 (86.1%)	0.29
Cardiovascular disease (+/-)	4 (15.4%)/22 (84.6%)	10 (12.7%)/69 (87.3%)	0.74
Respiratory disease (+/-)	2 (7.7%)/24 (92.3%)	10 (12.7%)/69 (87.3%)	0.73
Gastroenterological disease (+/-)	5 (19.2%)/21 (80.8%)	10 (12.7%)/69 (87.3%)	0.52
Renal disease (+/-)	5 (19.2%)/21 (80.8%)	7 (8.9%)/72 (91.1%)	0.17
Hematological disease (+/-)	1 (3.8%)/25 (96.2%)	2 (2.5%)/77 (97.5%)	1.00
Endocrinological disease (+/-)	2 (7.7%)/24 (92.3%)	10 (12.7%)/69 (87.3%)	0.73
Diabetes Mellitus (+/-)	7 (26.9%)/19 (73.1%)	13 (16.5%)/66 (83.5%)	0.26
Hypertension (+/-)	3 (11.5%)/23 (88.5%)	22 (27.8%)/57 (72.2%)	0.11

2. Preoperative status			

WBC (/mm^3^) **∗**	9450 (1100 - 37400)	8700 (1700 - 28800)	0.74
CRP (mg/dl) **∗**	13.3 (0.6 - 43.5)	12.9 (0.1 - 48.9)	0.39
SIRS (+/-)	16 (61.5%)/10 (38.5%)	38 (48.1%)/41 (51.9%)	0.26

3. Surgical factors			

The time from onset of symptoms to operation (> 12hr/≤12hr)	15 (57.7%)/11 (42.3%)	44 (55.7%)/35 (44.3%)	1.00
Perforation site (C-T/D-R)	10 (38.5%)/16 (61.5%)	19 (24.1%)/60 (75.9%)	0.21
Fecal peritonitis (+/-)	7 (26.9%)/19 (73.1%)	28 (35.4%)/51 (64.6%)	0.48

4. Postoperative status			

WBC on POD 1 (/mm^3^) **∗**	8400 (900 - 39900)	9100 (700 - 40300)	0.83
CRP on POD 1 (mg/dl) **∗**	15.2 (2.3 - 37.8)	15.7 (0.2 - 35.9)	0.68
Blood culture (+/-) ^††^	14 (63.6%)/8 (36.4%)	11 (32.1%)/45 (67.9%)	< 0.001
DIC (+/-)	13 (50.0%)/13 (50.0%)	21 (26.6%)/58 (73.4%)	0.03
PMX-DHP (+/-)	13 (50.0%)/13 (50.0%)	26 (32.9%)/53 (67.1%)	0.16

*∗* Median (min-max), ^†^ with some duplications, and ^††^ blood culture was carried out in 78 patients.

WBC: white blood cell. CRP: C-reactive protein. SIRS: systemic inflammatory response syndrome. C-T: Cecum-Transverse colon. D-R: Descending colon-rectum. POD: postoperative day. DIC: disseminated intravascular coagulation. PMX-DHP: polymyxin B-immobilized fiber column hemoperfusion.

**Table 5:** Clinicopathological factors in survivors and nonsurvivors among patients with connective tissue disease (CTD).

	Survivor group (n=21)	Non-survivor group (n=5)	p-value
1. Patients backgrounds			

Age **∗**	61 (38 - 83)	73 (28 - 95)	0.16
Gender (Male/Female)	5 (23.8%)/16 (76.2%)	2 (40.0%)/3 (60.0%)	0.59
Preoperative comorbidity^ †^			
Neurological disease (+/-)	1 (4.8%)/20 (95.2%)	0 (0%)/5 (100%)	1.00
Cardiovascular disease (+/-)	4 (19.0%)/17 (81.0%)	0 (0%)/5 (100%)	0.56
Respiratory disease (+/-)	2 (9.5%)/19 (90.5%)	0 (0%)/5 (100%)	1.00
Gastroenterological disease (+/-)	3 (14.3%)/18 (85.7%)	2 (40.0%)/3 (60.0%)	0.24
Renal disease (+/-)	4 (19.0%)/17 (81.0%)	1 (20.0%)/4 (80.0%)	1.00
Hematological disease (+/-)	1 (4.8%)/20 (95.2%)	0 (0%)/5 (100%)	1.00
Endocrinological disease (+/-)	2 (9.5%)/19 (90.5%)	0 (0%)/5 (100%)	1.00
Diabetes Mellitus (+/-)	5 (23.8%)/16 (76.2%)	2 (40.0%)/3 (60.0%)	0.59
Hypertension (+/-)	2 (9.5%)/19 (90.5%)	1 (20.0%)/4 (80.0%)	0.49

2. Preoperative status			

WBC (/mm^3^) **∗**	9500 (1600 - 18100)	7700 (1100 - 37400)	0.79
CRP (mg/dl) **∗**	9.7 (0.6 - 43.5)	16.9 (3.0 - 32.0)	0.72
SIRS (+/-)	12 (57.1%)/9 (42.9%)	4 (80.0%)/1 (20.0%)	0.62
Dose of steroids (mg/day) **∗**	15 (2 - 45)	11 (3 - 24)	0.43
Accumulated steroid dose (mg/month) **∗**	420 (60 - 750)	330 (60 - 881.5)	0.77
Duration of CTD (year) **∗**	13 (0.2 - 29)	1.8 (1.1 - 9.0)	0.055
Duration of steroid intake (year) **∗**	9.3 (0.04 - 29)	1.8 (0.8 – 9.0)	0.26
Steroid pulse therapy (+/-)	4 (19.0%)/17 (81.0%)	1 (20.0%)/4 (80.0%)	1.00
Immunosuppressive agent (+/-)	5 (23.8%)/16 (76.2%)	3 (60.0%)/2 (40.0%)	0.15

3. Surgical factors			

The time from onset of symptoms to operation (> 12hr/≤12hr)	13 (61.9%)/8 (38.1%)	2 (40.0%)/3 (60.0%)	0.33
Perforation site (C-T/D-R)	9 (42.9%)/12 (57.1%)	1 (20.0%)/4 (80.0%)	0.62
Fecal peritonitis (+/-)	3 (14.3%)/18 (85.7%)	4 (80.0%)/1 (20.0%)	0.01

4. Postoperative status			

WBC on POD 1 (/mm^3^) **∗**	9800 (2500 - 33200)	5600 (900 - 39900)	0.24
CRP on POD 1 (mg/dl) **∗**	15.2 (2.3 - 37.8)	12.2 (6.6 - 27.1)	0.77
Blood culture (+/-) ^††^	12 (63.2%)/7 (36.8%)	2 (66.7%)/1 (33.3%)	1.00
DIC (+/-)	10 (47.6%)/11 (52.4%)	3 (60.0%)/2 (40.0%)	1.00
PMX-DHP (+/-)	10 (47.6%)/11 (52.4%)	3 (60.0%)/2 (40.0%)	1.00
Postoperative dose of steroids (mg/day) **∗**	30 (5 - 86.5)	20 (10 - 45)	0.60

*∗* Median (min-max), ^†^ with some duplications, and^††^ blood culture was carried out in 22 patients.

WBC: white blood cell. CRP: C-reactive protein. SIRS: systemic inflammatory response syndrome. C-T: Cecum-Transverse colon. D-R: Descending colon-Rectum. POD: postoperative day. DIC: disseminated intravascular coagulation. PMX-DHP: polymyxin B-immobilized fiber column hemoperfusion.

**Table 6:** Prognostic factors for mortality within 28 days after surgery in patients with connective tissue disease (CTD).

	p-value	Odds ratio	95% Confidence interval
Fecal peritonitis	0.03	31.96	2.29 - 1675.22
Duration of CTD	0.12	0.79	0.59 - 1.07

**Table 7:** Detailed information for five patients who died within 28 days after surgery for colorectal perforation.

Age	Gender	Connective tissue disease (CTD)	Duration of CTD (year)	Preoperative dose of steroids (mg/day)	Perforation site	Cause of perforation	Peritonitis	Postoperative survival period (day)
28	Male	Adult Still disease	8.0	9	S	Ulcer	Fecal	5
71	Female	Polyarteritis nodosa	1.1	24	S	Diverticulum	Fecal	17
88	Female	Rheumatoid arthritis	1.1	15	S	Diverticulum	Fecal	22
95	Male	Polymyalgia rheumatica	9.0	11	D	Idiopathic	Non-fecal	25
73	Female	Systemic sclerosis	1.8	3	A	Colorectal cancer	Fecal	2

## Data Availability

Our data in this manuscript were obtained from patients who underwent surgery for colorectal perforation in Juntendo University Hospital between January 2003 and September 2017. If the authors are asked to show data regarding this manuscript, they can show them.
